# Hematocrit Sign Elucidates Cause of Abdominal Pain

**DOI:** 10.24908/pocus.v6i1.14751

**Published:** 2021-04-22

**Authors:** Jeffrey Lam, Steven Montague

**Affiliations:** 1 Department of Medicine, Division of General Internal Medicine, Queen's University Kingston, Ontario Canada

**Keywords:** POCUS, hematocrit sign, kidney disease

## Abstract

A 78-year-old male with chronic kidney disease on peritoneal dialysis developed unprovoked bilateral pulmonary embolisms. He was started on IV unfractionated heparin, but shortly thereafter developed severe pain and a small firm abdominal nodule near his dialysis catheter site. The diagnosis was unknown, and the initial plan was watchful waiting, until point-of-care ultrasound (POCUS) was used. POCUS revealed an ovoid mass with hyperdensity in the gravity dependent regions with spontaneous movement. This appearance was classic for the hematocrit sign. When combined with the clinical presentation, this was concerning for a rectus sheath hematoma. An urgent CT of the abdomen confirmed this several hours later. POCUS allowed for rapid bedside diagnosis, which expedited appropriate care in a potentially life-threatening situation.

## Case File

A 78-year-old male with Stage V chronic kidney disease on peritoneal dialysis (PD), and numerous other cardiovascular co-morbidities, presented to the Emergency Department with a 4-day history of dyspnea. A ventilation/perfusion scintigraphy revealed bilateral unprovoked pulmonary emboli (PE). The patient was started on warfarin and IV unfractionated heparin as bridging anticoagulation. Three days after starting therapeutic anticoagulation, the patient developed severe pain located 5cm adjacent to his PD catheter insertion site. Palpation revealed a firm, 2cm, well-circumscribed, tender subcutaneous mass. The etiology was unclear, and the initial plan was to follow clinically. 

**Figure 1:**
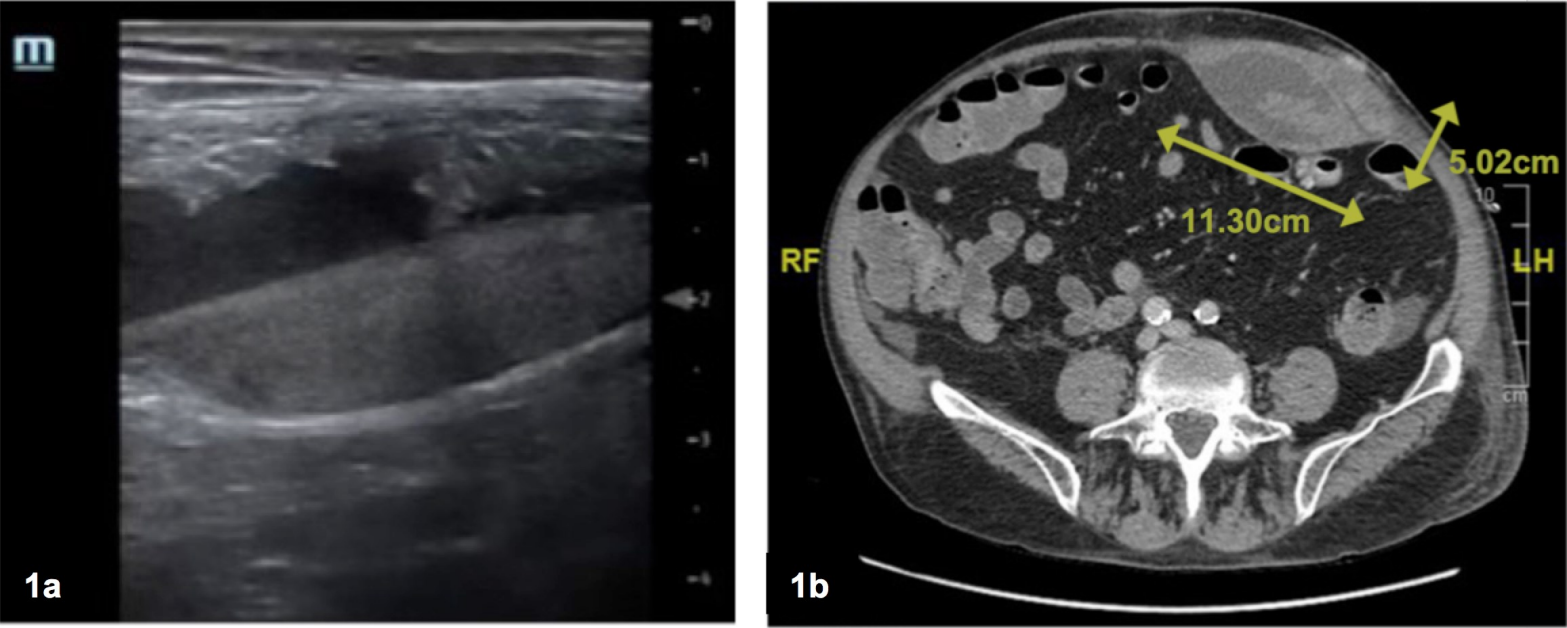
a) Point of Care Ultrasound of Left Lower Abdominal Quadrant mass showing the hematocrit sign. b) CT abdomen and pelvis showing a 11x11x5cm rectus sheath hematoma.

Point-of-care ultrasound (POCUS) was performed to screen for an underlying etiology. POCUS revealed an unexpected finding. Within the subcutaneous tissue, outside of the peritoneum, there was a discrete ovoid visualized (Figure 1a, online Video S1). This structure was hyperechoic in the gravity dependent portion. Additionally, there appeared to be spontaneous movement within the ovoid. Taken together, these findings represent active extravasation forming a rectus sheath hematoma. The fluid-fluid layer, with the gravity dependent fluid being hyperechoic, is known as the hematocrit sign, which was the hallmark POCUS finding visualized. 

Rectus sheath hematoma (RSH) is an uncommon clinical condition whereby blood accumulates in the rectus abdominus muscle sheath as a result of damaged muscle fibres or torn epigastric vessels 1. This is typically due to blunt abdominal trauma, or overly forceful muscle contraction, but can occur spontaneously particularly if patients are on anticoagulation 2. Patients commonly present with acute abdominal pain and a palpable abdominal mass but may also have nausea, vomiting, fevers, and chills 2, 3. A high index of suspicion is required as abdominal wall pathology can mimic other causes of acute abdomen, making it a frequently overlooked diagnosis 4. Appropriate investigations include monitoring for decreasing hemoglobin and imaging (ultrasound or CT of abdomen) 2. Most patients with RSH are managed conservatively as they are often self-limiting but in hemodynamically unstable patients, embolization or surgical ligation may be required 2. Complications associated with RSH include muscle necrosis, hypovolemic shock, abdominal compartment syndrome, myocardial infarction, and death 3, 5.

The hematocrit sign is an imaging finding frequently associated with superficial hematomas or hemothorax 6, 7, 8. The appearance of the hematoma varies depending on the duration of bleeding. Hematomas may appear heterogeneously hypoechoic initially and become increasingly hyperechoic over time 6. The hematocrit sign (Figure 1a, Video S1) occurs as coagulated cells and debris collect in dependent areas due to gravity, forming a distinct line of separation between liquid and cellular components of blood 7, 9. There is no mimic for this sonographic finding; although the main differential could include a subcutaneous abscess, the contents of a subcutaneous abscess would not typically display a well defined hyperechoic layer as seen in the hematocrit sign.

As the patient developed acute abdominal pain, the underlying cause was initially suspected to be due to peritoneal irritation from PD or a hernia. POCUS significantly expedited the diagnosis, allowing for rapid cessation of heparin, and initiation of close monitoring. Anticoagulation was indicated for his large unprovoked bilateral PE, but due to the RSH the decision was made to stop the IV heparin, and continue warfarin targeting an INR of 2.0. An urgent abdominal CT several hours later confirmed the diagnosis, showing at that time an 11x11x5cm rectus sheath hematoma in the left lower quadrant (Figure 1b). The patient required a transfusion of three units of packed red blood cells but otherwise improved clinically with no further complications. 

## Statement of Ethics

The authors certify that informed consent was obtained from the patient. The patient has consented to the use of images, video clips, and information regarding his condition and treatment to be published within the journal. 

## Disclosures

The authors have no conflicts of interest to declare.

## Supplementary Material

 Video S1 POCUS showing the hematocrit sign, with hyperdensity in the gravity dependent regions and spontaneous movement.
